# Comparison of the effects of twice-daily exenatide and insulin on carotid intima-media thickness in type 2 diabetes mellitus patients: a 52-week randomized, open-label, controlled trial

**DOI:** 10.1186/s12933-020-01014-7

**Published:** 2020-04-25

**Authors:** Jie Zhang, Tong-Zhang Xian, Ming-Xiao Wu, Chen Li, Qi Pan, Li-Xin Guo

**Affiliations:** 1 Department of Endocrinology, Beijing Hospital, National Center of Gerontology; Institute of Geriatric Medicine, Chinese Academy of Medical Sciences, Graduate School of Peking Union Medical College, No. 1 Dahua Road, Dong Dan, Beijing, 100730 P. R. China; 2 Department of Endocrinology, Beijing Hospital, National Center of Gerontology; Institute of Geriatric Medicine, Chinese Academy of Medical Sciences, No. 1 Dahua Road, Dong Dan, Beijing, 100730 P. R. China; 3 Department of Ultrasound, Beijing Hospital, National Center of Gerontology, Institute of Geriatric Medicine, Chinese Academy of Medical Sciences, No. 1 Dahua Road, Dong Dan, Beijing, 100730 P. R. China

**Keywords:** Carotid intima-media thickness, Exenatide, Insulin, Low-density lipoprotein cholesterol

## Abstract

**Background:**

Exenatide, a glucagon like peptide 1 analog, has been suggested to reduce the cardiovascular disease risk factors, such as body weight, blood pressure and subclinical atherosclerosis in patients with type 2 diabetes mellitus (T2DM). This was the first randomized, open-label, controlled trial to compare the effects of exenatide versus insulin on subclinical atherosclerosis, as assessed by carotid-intima media thickness (CIMT), in patients with T2DM.

**Methods:**

A total of 66 patients with T2DM admitted from March 10, 2015 to June 20, 2017 in the Department of Endocrinology, Beijing Hospital were randomized to receive twice-daily exenatide or aspartate 70/30 insulin for 52 weeks. The primary endpoint was change from baseline in CIMT, and secondary endpoints included changes at week 52 from baseline in body weight, glycemic markers, lipid metabolism markers, blood pressure, C-reactive protein, fibrinogen, 8-hydroxydeoxyguanosine, irisin, and brain natriuretic peptide.

**Results:**

Exenatide more significantly reduced the CIMT from baseline compared with insulin after 52 weeks, with a mean difference of − 0.14 mm (95% interval confidence: − 0.25, − 0.02; P = 0.016). Weight and body mass index were both significantly reduced in the exenatide group over 52 weeks. Exenatide reduced total lipoprotein and low-density lipoprotein cholesterol levels more significantly than insulin at weeks 16 and 40. Correlation analyses showed that CIMT was positively correlated with low-density lipoprotein cholesterol.

**Conclusions:**

Twice-daily exenatide could prevent atherosclerosis progression in patients with T2DM over a 52-week treatment period compared with insulin therapy.

*Trial registration* Chinese Clinical Trial Registry ChiCTR-1800015658

## Background

Type 2 diabetes mellitus (T2DM) is a chronic and progressive metabolic disease characterized by hyperglycemia due to the defects of insulin secretion and/or action [1]. The incidence rates of atherosclerotic cardiovascular disease and peripheral arterial disease are elevated among patients with T2DM [2, 3], and some large-scale studies have shown that exogenous insulin therapy may worsen cardiovascular outcomes in T2DM patients [4, 5].

Human glucagon like peptide 1 (GLP-1) receptor agonists have been developed to augment insulin secretion and inhibit glucagon secretion to control glycemic excursions [6]. It has been reported that fasting total GLP-1 is significantly negatively correlated with CIMT in male T2DM patients [7]. Exenatide twice-daily, a short-acting GLP-1 analog, has been approved for the treatment of T2DM as a GLP-1 receptor agonist [8, 9]. The EXSCEL trial demonstrated that the incidence of major adverse cardiovascular events was not significantly different between patients treated with extended-release exenatide and placebo [10]. In addition, exenatide delays gastric emptying, inhibits food intake, and limits weight gain [6, 11]. A retrospective study revealed that twice-daily exenatide treatment reduced the risks of cardiovascular diseases in patients with T2DM [12]. Exenatide twice-daily showed significant improvement in cardiovascular risk markers including weight, high-density lipoprotein-cholesterol (HDL-C) level and high-sensitivity C-reactive protein (hsCRP) level [13, 14]. Carotid intima-media thickness (CIMT) is a surrogate marker for subclinical atherosclerosis worldwide using simple and noninvasive B-mode carotid ultrasound [15, 16]. Liraglutide, also a GLP-1 analog, improved the CIMT in T2DM patients in an 18-month prospective study [17]. Exenatide once weekly significantly improved fasting glycemia, glycosylated hemoglobin (HbA1c), body mass index (BMI), lipid profile and CIMT in patients with T2DM in an 8-month prospective study [18]. However, effects of exenatide twice-daily compared with insulin therapy on CIMT in T2DM patients have not been evaluated by a randomized trial so far. In the present study, we aimed to compare the efficacy of exenatide to that of insulin for improving atherosclerosis markers (e.g., CIMT, hsCRP, fibrinogen, and 8-hydroxydeoxyguanosine [8-OHdG]), body weight, blood pressure, glycemic control and dyslipidemia (e.g., HbA1c, fasting plasma glucose level, and lipid profile) in a randomized, open-label, controlled trial in T2DM patients.

## Methods

### Study population

This single-center randomized, open-label, controlled trial was performed in the Department of Endocrinology, Beijing Hospital and was performed in accordance with the Declaration of Helsinki. The research ethics committee of Beijing Hospital reviewed and approved the study protocol before the enrollment of patients (No. 2013 BJYYEC-017A-03). All participants were informed of the details of the study and signed the corresponding consent forms.

Patients with T2DM admitted from March 10, 2015 to June 20, 2017 were screened for enrollment. T2DM patients were included if they met the following criteria: (1) diagnosed with T2DM according to the 1999 WHO criteria; (2) aged between 20 and 75 years; (3) glucose control was not satisfactory with HbA1c level between 7.5 and 11%; (4) had taken at least two oral hypoglycemic drugs with higher than 1/2 of the maximum dose for at least 3 months. Patients were excluded if they had any of the following clinical conditions: type 1 diabetes; > 75% stenosis of any segment of the carotid artery by high frequency B mode ultrasound; an acute cardiovascular event within 30 days prior to randomization; currently planned cardiovascular, carotid or peripheral artery revascularization or cardiac valvular surgery; previous use of insulin or exenatide more than 1 month; an alanine aminotransferase (ALT) or aspartate aminotransferase (AST) level > 2.5 times the upper limit of normal range; serum creatinine concentration ≥ 133 µmol/L for males or ≥ 106 µmol/L for females; history of pancreatitis; currently participating in or having completed another clinical trial within 3 months; or positive for human urinary chorionic gonadotropin or could not adopt a contraceptive method during the study.

### Study design

Eligible patients were randomized 1:1 to receive exenatide or insulin aspart 70/30 using computer codes, and patients were stratified according to the severity of disease (7.5% and 9%). Patients allocated to the exenatide group were given exenatide 5 µg twice-a-day (administration 60 min before breakfast and dinner) subcutaneously, and the dose was increased to 10 µg twice-a-day after 4 weeks. Patients allocated to the insulin group were given insulin aspartate 70/30 subcutaneously. The initial dose of insulin was 0.2–0.4 IU/kg per day and was titrated according to self-monitoring blood glucose and HbA1c detected every 12 weeks. The titration of insulin could be completed in a visit or a telephone follow-up by the investigators following the protocol shown in Additional file 1: Table S1. All of the included patients were free to take hypoglycemic drugs except for sulfonylureas and nateglinide drugs. All the participants were educated on a suitable diet and exercise.

The primary outcome of the study was the change in CIMT from baseline to week 52. The secondary objectives included change from baseline to week 52 in atherosclerosis markers (e.g., hsCRP, fibrinogen and 8-OHdG), body weight, blood pressure (diastolic and systolic blood pressure), glycemic control (e.g., HbA1c, fasting plasma glucose level) and dyslipidemia (e.g., total cholesterol [TC], HDL-C, triglyceride [TG], and low-density lipoprotein cholesterol [LDL-C]).

The CIMT was measured using a Philips iU22 Color Doppler ultrasound (Bothell, WA, USA). Two physicians from the Ultrasound Department of Beijing Hospital performed the ultrasound examination after receiving uniform training. Briefly, the patients assumed the supine position, and the IMTs of the bilateral carotid arteries were measured. Measurements were made for three segments: the carotid artery to the dilated portion of the carotid artery, the enlargement of the carotid artery, and the internal carotid artery, which was within a 1-cm range from the distal portion of the carotid artery to the dilated portion of the carotid artery. The IMT of the posterior wall was measured. The maximum IMT values for the posterior wall of the carotid arteries within the three segments were measured, and the bilateral maximum mean value was used for statistical analysis. Each participant was examined by the same sonographer using the same equipment throughout all visits.

### Serum sample analysis

TG, TC, LDL, and HDL were assessed using standard commercial oxidase method (TC intra-assay coefficient of variation [CV] 0.8%, inter-assay CV 2.22%; TG intra-assay CV 0.48%, inter-assay CV 1.46%; LDL intra-assay CV 0.71%, inter-assay CV 1.59%; and HDL intra-assay CV 0.83%, inter-assay CV 1.13%;). A kit from Trinity Biotech was used for assessing HA1c (intra-assay CV < 2%, inter-assay CV < 3%), and the glucose oxidase method was used for measuring FPG (intra-assay CV 0.43%, inter-assay CV 1.81%). Hs-CRP was measured by immune turbidimetric assay (intra-assay CV 4%, inter-assay CV 7%). The evaluation of the fibrinogen level was performed with Kanto Kagaku’s assay (intra-assay CV 2.8%, inter-assay CV 5.5%). Determinations of 8-OHdG, Irisin, and brain natriuretic peptide (BNP) was carried out by ELISA (8-OHdG intra-assay CV 5.7%, inter-assay CV 7.2%; Irisin intra-assay CV 5.4%, inter-assay CV 6.7%; and BNP intra-assay CV 5.2%, inter-assay CV 6.3%). All measurements were performed according to the manufacturer’s instructions.

### Statistical analysis

All analyzes were performed with SAS V.9.10 software (SAS, Cary, NC, USA). Regarding the sample size estimation, we assumed a 0.15 mm difference in the CIMT between the exenatide and insulin group based on previous liraglutide and DPP-IV studies [19, 20]. Considering a standard deviation of 0.2 mm, 28 patients per treatment group were needed for the primary endpoint analysis to warrant a power of 80% with a two-sided significance level of 0.05. Assuming a drop-out rate of 15%, the sample size needed for each group would be 33, for a total sample size of 66 patients.

The full analysis set was used for statistical analysis. For the primary endpoints, the least-squares mean change from baseline to 52 weeks and associated 95% confidence intervals and P values for exenatide versus insulin were derived from a mixed model for repeated measures with age, sex, duration of T2DM and CIMT at baseline as fixed covariates. Normally distributed data are expressed as means and standard deviations, and the t-test was used for comparison. Skewed data were compared with a nonparametric test. The count data are expressed as proportions, and the frequencies were compared with a Chi-square test. A P < 0.05 indicated a statistically significant difference. All statistical analyses were carried out with IBM SPSS statistical software V.22 for Windows (IBM Corp., Armonk, NY, USA).

## Results

**Fig. 1:**
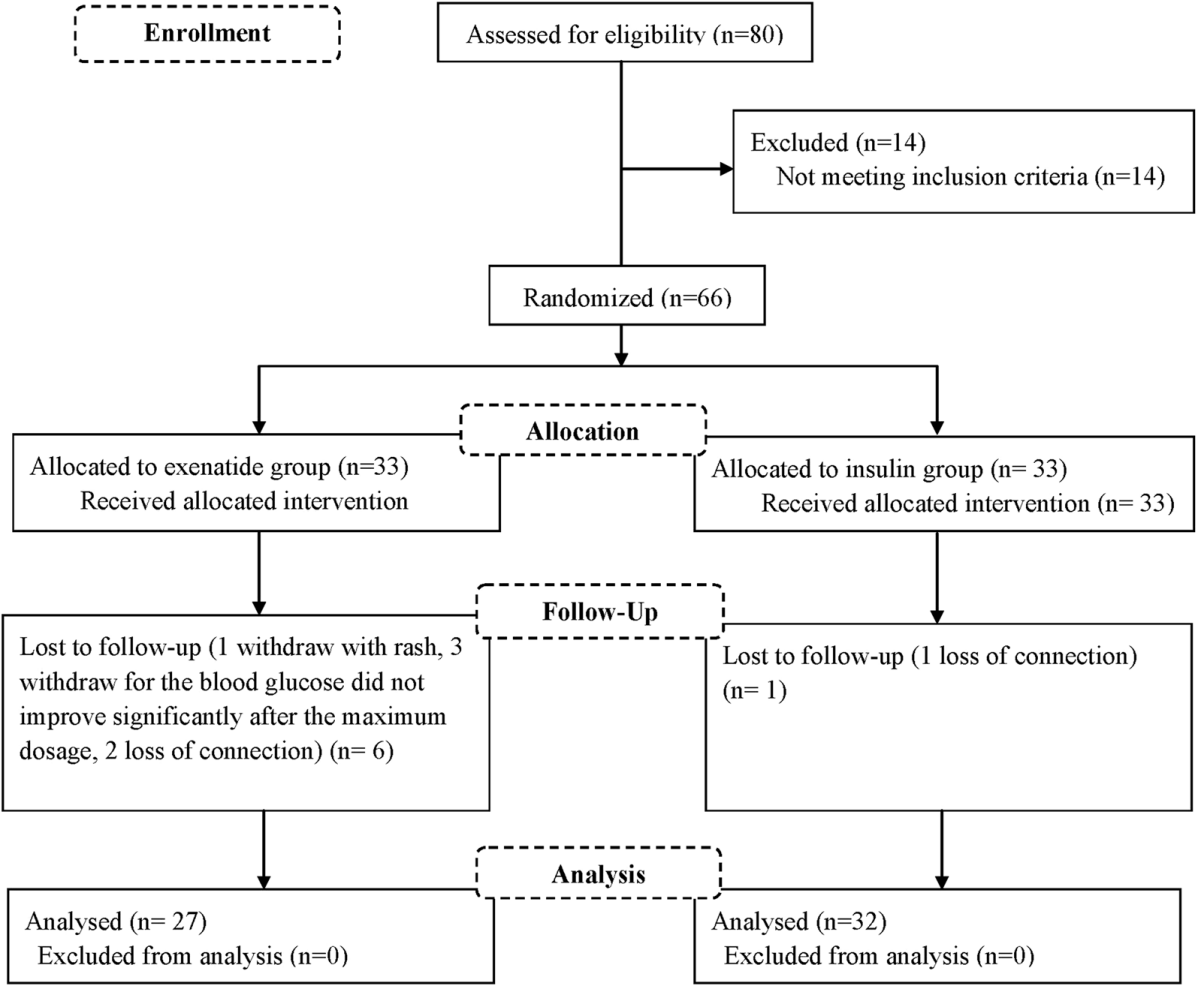
Flow diagram of the study

Overall, 80 patients were screened and 14 were excluded for not meeting the inclusion criteria. The remaining 66 patients were randomized into the exenatide group or insulin group at a 1:1 ratio. Finally, 27 patients treated with exenatide and 32 patients treated with insulin were included in the analysis. The baseline characteristics of the patients are shown in Table 1. Patients in the two groups were well balanced for most of the baseline characteristics except for gender and diastolic blood pressure. A total of 6 patients did not complete the study due to rash (n = 1), non-response to study drug (n = 3) or loss to follow-up (n = 2) in the exenatide group. In the insulin group, one patient was lost to follow-up (Fig. 1).

**Table 1:** Demographic and baseline characteristics

	Exenatide group (n = 27)	Insulin group (n = 32)	P value
Sex (male/female)	19/8	14/18	0.038
Age (years)	58.85 ± 12.54	58.03 ± 13.32	0.657
Weight (kg)	68.68 ± 11.95	66.30 ± 11.42	0.438
BMI (kg/m^2^)	23.64 ± 2.86	24.36 ± 2.52	0.313
Diabetes durations (years)	6.59 ± 5.32	7.81 ± 6.02	0.417
Systolic pressure (mmHg)	127.03 ± 16.48	125.46 ± 15.04	0.704
Diastolic pressure (mmHg)	80.18 ± 9.65	75.15 ± 9.11	0.044
Fasting glucose (mmol/L)	10.38 ± 2.95	10.81 ± 2.52	0.544
HbA1c (%)	8.67 ± 1.03	8.32 ± 0.96	0.195
Total cholesterol (mmol/L)	4.69 ± 1.40	4.62 ± 0.86	0.826
Triglyceride (mmol/L)	2.18 ± 1.50	2.10 ± 1.49	0.834
Low-density lipoprotein cholesterol (mmol/L)	2.18 ± 1.50	2.10 ± 1.49	0.423
High-density lipoprotein cholesterol (mmol/L)	1.06 ± 0.31	1.13 ± 0.24	0.335
Uric acid (µmol/L)	307.51 ± 102.65	288.53 ± 80.16	0.428
Hypersensitive C reactive protein (mg/L)	2.93 ± 3.15	2.02 ± 1.88	0.170
Fibrinogen (g/L)	2.88 ± 0.65	2.92 ± 0.54	0.810
8-OHdG (ng/mL)	6.60 ± 0.49	6.59 ± 0.67	0.941
Statins (n)	4	11	0.086
Aspirin (n)	5	9	0.388
Calcium antagonists (n)	6	9	0.604
ARB/ACEI (n)	9	11	0.933
Beta blockers (n)	4	2	0.278

*BMI* body mass index, *HbA1c* glycosylated hemoglobin, *8-OHdG* 8-hydroxydeoxyguanosine

**Fig. 2:**
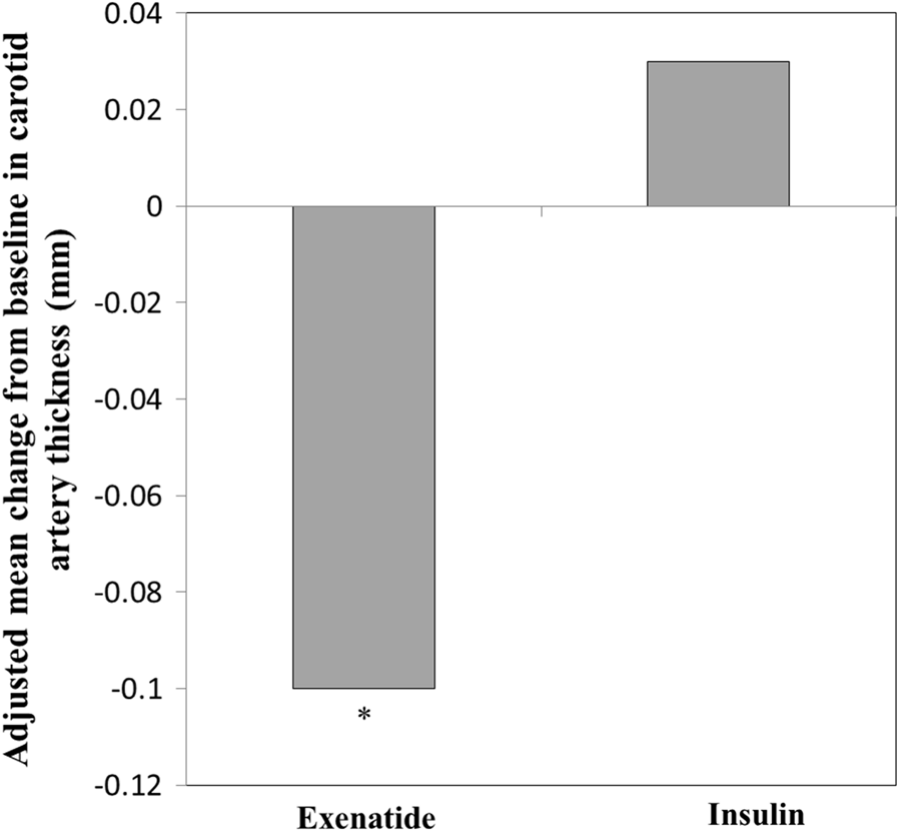
Adjusted mean change from baseline in carotid artery thickness after 52 weeks in the exenatide or insulin group. *P < 0.05 (compared to insulin group). Data are adjusted least-square mean difference

**Fig. 3:**
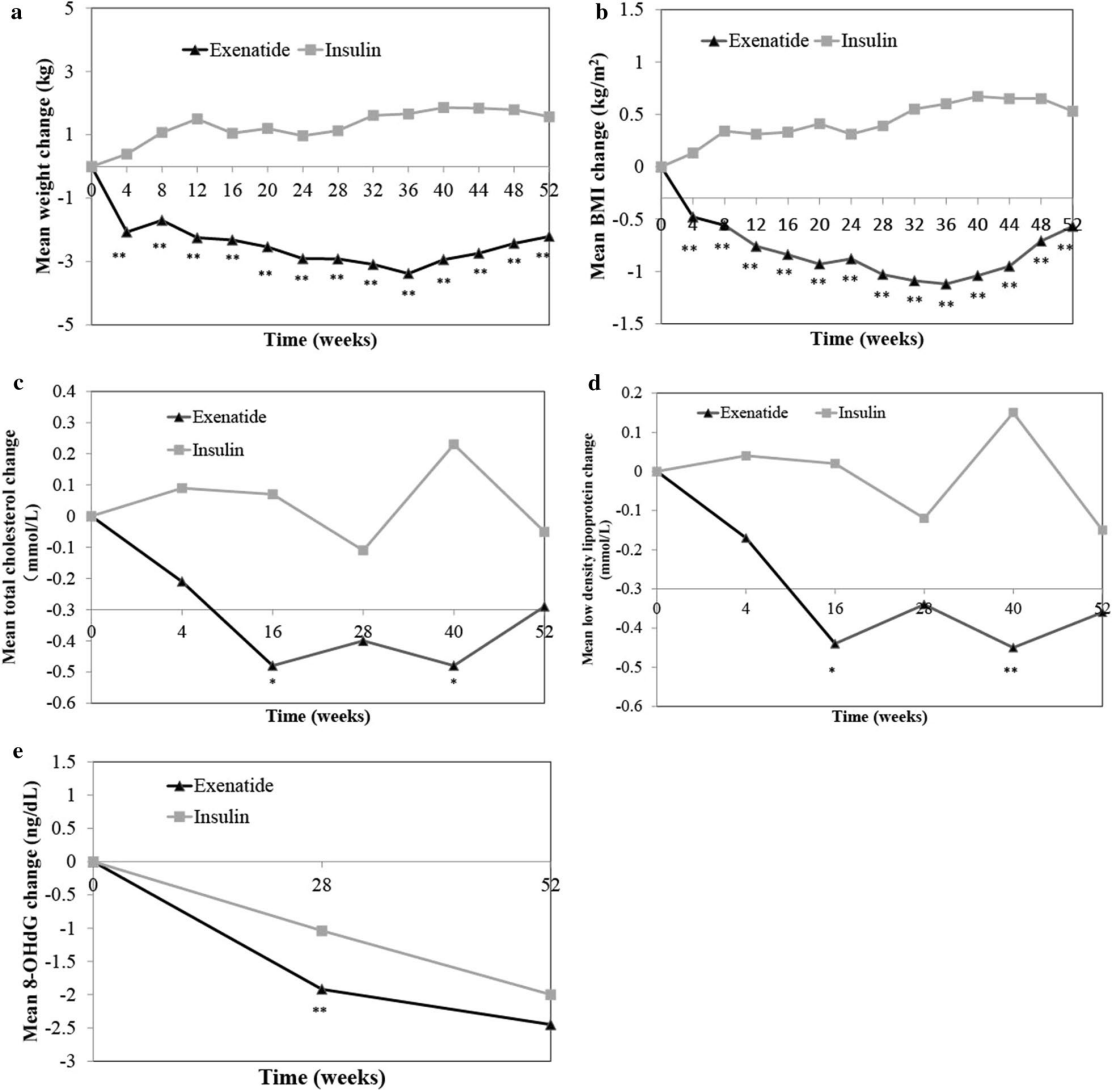
Changes in mean weight (**a**), BMI (**b**), total lipoprotein cholesterol (**c**), low density lipoprotein cholesterol (**d**), and 8-OHdG (**e**) from baseline to 52 weeks in the exenatide and insulin groups. *P < 0.05; **P < 0.01 (compared to insulin group)

Exenatide more significantly reduced CIMT than did insulin, with a mean difference of − 0.14 mm (95% interval confidence: − 0.25, − 0.02) after 52 weeks (P = 0.016; Fig. 2). Additionally, exenatide more significantly reduced body weight than did insulin at each time point (Fig. 3a, P < 0.01), with a mean difference of − 2.21 kg after 52 weeks. Similarly, exenatide reduced BMI significantly more than insulin at each time point (Fig. 3b, P < 0.01), with a mean difference of − 0.5 kg/m^2^ after 52 weeks.

The diastolic and systolic blood pressures were both not significantly reduced in either the exenatide or insulin group after 52 weeks compared with baseline values, with no significant differences between the two groups for either diastolic or systolic blood pressure (Table 2).

**Table 2:** Primary and secondary endpoints after 52 weeks (FAS)

	Exenatide group (n = 27)	Insulin group (n = 32)	P^1^ value
Primary endpoint			
Carotid intima-media thickness, adjusted mean change (95% CI) (mm)	− 0.10 (− 0.18, − 0.02)	0.03 (− 0.03, 0.11)	0.016
Secondary endpoints			
Weight (kg)	− 2.21 ± 3.43**	1.57 ± 2.96**	< 0.01
Systolic pressure (mmHg)	− 1.85 ± 14.35	2.25 ± 18.74	0.357
Diastolic blood pressure (mmHg)	− 4.25 ± 11.66	− 2.12 ± 10.42	0.461
HbA1c (%)	− 1.31 ± 1.42**	− 1.09 ± 1.42**	0.561
Fasting blood glucose (mmol/L)	− 0.79 ± 3.49	− 2.39 ± 3.40**	0.085
Total cholesterol (mmol/L)	− 0.29 ± 1.20	− 0.05 ± 1.04	0.433
Triglyceride (mmol/L)	− 0.17 ± 1.49	− 0.08 ± 1.55	0.826
Low density lipoprotein (mmol/L)	− 0.36 ± 0.83*	− 0.15 ± 0.87	0.365
High density lipoprotein (mmol/L)	0.02 ± 0.16	− 0.00 ± 0.15	0.416
High sensitivity C-reactive protein (mg/L)	0.39 ± 2.55	1.66 ± 4.55*	0.183
Fibrinogen (g/L)	0.35 ± 0.44**	0.48 ± 0.48**	0.290
8-OHdG, ng/dL (ng/mL)	− 2.45 ± 0.74**	− 2.00 ± 0.75**	0.058
Exploratory endpoints			
Irisin (pg/mL)	26.01 ± 7.86**	24.91 ± 8.65**	0.665
Brain natriuretic peptide (pg/mL)	− 103.55 ± 39.47**	− 84.11 ± 27.95**	0.065

Data are presented as mean change ± standard deviation unless otherwise noted

*8-OHdG* 8-hydroxydeoxyguanosine

** and * were represented as significantly different from baseline with P < 0.01 and P < 0.05, respectively

^1^P value is shown for mean change comparison between exenatide and insulin group

Although exenatide significantly reduced HbA1c from baseline (P < 0.01), this reduction was not significantly greater than that achieved with insulin (Table 2). For fasting plasma glucose level change, no significant difference was observed between the two groups (Table 2).

Total cholesterol and LDL-C levels were both reduced more significantly in the exenatide group than in the insulin group at weeks 16 and 40 (Fig. 3c, d, P < 0.05 and P < 0.01, respectively). However, there was no significant difference between the two groups at week 52 (Table 2).

Changes in the hsCRP and fibrinogen levels from baseline were both not significantly different between the two groups after 52 weeks (Table 2). Both exenatide and insulin significantly reduced the 8-OHdG level from baseline to week 52 (Table 2). Exenatide was associated with a more significant reduction in the 8-OHdG level compared with insulin at week 28 (P < 0.01, Fig. 3e), while no significant difference was observed at week 52 (Table 2). The irisin level was increased both in the exenatide and insulin groups after 52 weeks (P < 0.01). The brain natriuretic peptide level was decreased in both the exenatide and insulin groups after 52 weeks. However, no significant difference was observed between the exenatide and insulin group for the irisin or brain natriuretic peptide (Table 2).

We further performed a correlation analysis to assess the association between CIMT and other markers in this study and found CIMT was positively correlated with LDL-C (r = 0.441,P = 0.021) and Fibrinogen (r = 0.605, P < 0.01). Hypoglycemia occurred in one patient treated with exenatide and five patients treated with insulin. No severe hypoglycemia events were reported in the trial.

## Discussion

An observational study for multi-center (71 centers) demonstrated that 20 weeks of treatment with short-acting exenatide was well tolerated and showed a significant body weight and glucose reduction in T2DM patients whose glycemia had been inadequately controlled with oral hypoglycemic agents [21]. Once-weekly exenatide resulted in a nominal 9% relative reduction in major adverse cardiovascular events and a 14% relative reduction in all-cause mortality compared to placebo in T2DM with and without known cardiovascular disease [22], whereas dipeptidyl peptidase-4 inhibitors had no effect on cardiovascular risk outcomes but increased risks of acute pancreatitis and hypoglycemia [23]. Another study showed that the use of dipeptidyl peptidase-4 inhibitors were associated with a reduced risk of heart failure hospitalization compared to GLP-1RAs [24]. Moreover, postoperative exenatide did not provide any additional cardioprotective effect compared to intravenous insulin in coronary artery bypass grafting patients [25]. The effects of GLP-1RA on the cardiovascular risk are still controversial. In this randomized controlled trial, for the first time, exenatide twice-daily more significantly reduced the CIMT in T2DM patients compared with insulin therapy over 52 weeks. To our knowledge, this is the first randomized study to show improvement in subclinical atherosclerosis, as assessed by CIMT, in patients with T2DM over a long period of 52 weeks.

In this study, exenatide improved the surrogate atherosclerotic marker CIMT in T2DM patients. The results were consistent with one prospective study for exenatide once weekly, which reported an improvement in CIMT in T2DM patients after 8 months of treatment [18]. Recently, several studies have investigated the effects of anti-diabetic agents, especially for GLP-1 receptor agonist, on CIMT in T2DM patients [17–19]. An observational study showed that exenatide twice-daily improved another surrogate atherosclerotic marker, arterial dilation, in patients with T2DM [26].

The weight and BMI were both decreased after exenatide treatment for 52 weeks, whereas they were increased after insulin treatment for 52 weeks. The reductions in both weight and BMI were significantly different between the exenatide and insulin groups. The glucose-lowering effect of exenatide was equal to that of premixed insulin used in our study, as evidenced by the non-significant difference in the HbA1c level between the two groups. These results are consistent with previous studies showing a similar decrease in HbA1c between exenatide and insulin treatment in T2DM patients, and exenatide-treated patients lost weight while insulin-treated patients gain weight [27, 28]. In addition, we observed that FPG was not significantly reduced at week 52 from baseline in the exenatide group, whereas it was significantly reduced with insulin treatment. It has been reported that exenatide supports a modest reduction in FBG in T2DM patients while insulin predominantly affects the FBG [11, 29]. Taken together, these results showed that exenatide might not be inferior to insulin in glucose-lowering activity and is superior to insulin in reducing body weight.

Interestingly, we found significant associations between the change in CIMT and LDL-C and fibrinogen concentrations by correlation analysis. These results were not consistent with a previous study [18], which showed no significant correlation between the CIMT and LDL-C level. However, in liraglutide-treated T2DM patients with metabolic syndrome, CIMT was significantly correlated with the TG level [17]. It has been reported that the fibrinogen concentration increases with the development and progression of T2DM [30]. However, neither exenatide nor insulin could reduce the fibrinogen concentration in our study. We also found that exenatide reduced the total cholesterol and LDL-C levels more significantly than did insulin at weeks 16 and 40. The total cholesterol and LDL-C levels were both reported to be significantly related with cardiovascular disease and atherosclerosis risk [31–33]. Exenatide [34] could reduce the serum total cholesterol level by about 5%, TG level by about 12%, and LDL-C level by about 6%, while increasing the HDL-C level by about 24% in T2DM patients for at least 3 years. There were no significant differences between these two groups in total cholesterol and LDL-C level at week 52. However, the average values for total cholesterol and LDL-C were both in normal range at baseline, which may explain lack of effect of exenatide on these cholesterol levels. Thus, the effect of exenatide on total cholesterol and LDL-C levels remains to be investigated in future studies, preferably in patients with hypercholesteremia.

The diastolic and systolic blood pressures were both not significantly reduced in the exenatide group after 52 weeks compared with baseline values, with no significant differences between the exenatide and insulin groups for either diastolic or systolic blood pressure. Notably, the systolic blood pressure was increased in the insulin group after 52 weeks of treatment. A post hoc analysis showed that the exenatide twice-daily dose did not affect blood pressure in T2DM patients [35], while another previous study reported that exenatide could reduce the diastolic and systolic blood pressures in T2DM patients [14]. In our study, the lack of efficacy of exenatide on blood pressure compared with insulin may because the diastolic or systolic blood pressure of most patients at baseline was well regulated.

Additionally, we found that exenatide was associated with a more remarkable reduction in 8-OHdG, a marker of oxidative stress to DNA and increased risk of atherosclerosis [36], compared with insulin. Taken together, our results suggest a potential role for exenatide in preventing atherosclerosis progression in T2DM patients.

Our study has some limitations. First, this single-center study did not have enough power to evaluate the influences of exenatide on some metabolic outcomes. Second, the effects of exenatide on clinical outcomes, particularly on cardiovascular outcomes, should be further compared with those of insulin. Finally, study participants were free to use metformin, and whether metformin and exenatide combination therapy affects the levels of metabolic and atherosclerotic markers deserves further investigation.

## Conclusions

In conclusion, exenatide inhibits atherosclerotic progression to delay the development of cardiovascular disease in patients with T2DM compared to insulin therapy, in addition to its benefits of glucose-lowering, body weight control, and dyslipidemia improvement.

## Supplementary information

**Additional file 1: Table S1.** Titration protocol for insulin in patients with type 2 diabetes mellitus.

## Data Availability

The datasets generated and analyzed during the current study are available from the corresponding author on reasonable request.

## Abbreviations

T2DM: Type 2 diabetes mellitus
CIMT: Carotid intima-media thickness
GLP-1: Glucagon like peptide 1
8-OHdG: 8-Hydroxydeoxyguanosine
ARB: Angiotensin receptor blocker
ACEI: Angiotensin I Converting Enzyme Inhibitor
