# Supplementary material for: Comparison of the effects of twice-daily exenatide and insulin on carotid intima-media thickness in type 2 diabetes mellitus patients: a 52-week randomized, open-label, controlled trial
Source: Cardiovasc Diabetol. 2020 Apr 25;19:48. doi: 10.1186/s12933-020-01014-7 (PMC7183674; doi:10.1186/s12933-020-01014-7)
Supplement: Supplementary file 1 — Additional file 1: Table S1. Titration protocol for insulin in patients with type 2 diabetes mellitus. [file 12933_2020_1014_MOESM1_ESM.doc]

**Additional file 1: Table S1 Titration protocol for insulin in patients with type 2 diabetes mellitus.**

| **Pre-dinner SMBG (mmol/L)** | **Insulin dose adjustment** |
| --- | --- |
| ≤4.3 | -2 units |
| 4.4-7.0 | No adjustment |
| 7.1-8.5 | +2 units |
| 8.6-11.1 | +4 units |
| ≥11.1 | +6 units |

SMBG, self-monitoring of blood glucose.
